# Enzyme‐based sensors: An annotated selection of World Wide Web sites relevant to the topics in *microbial biotechnology*

**DOI:** 10.1111/1751-7915.12364

**Published:** 2016-04-18

**Authors:** Lawrence P. Wackett

**Affiliations:** ^1^ Department of Biochemistry, Molecular Biology & BiophysicsBioTechnology InstituteUniversity of MinnesotaSt. PaulMN55108USA

## Enzymatic biosensors

### http://www.mdpi.com/journal/sensors/special_issues/enzymatic-biosensors

This address links to a special issue of the journal *Sensors* that is focused specifically on enzyme‐based sensors.

## Biosensors: Wikipedia

### https://en.wikipedia.org/wiki/Biosensor

This general overview describes different types of biosensors, one type of which is based on enzyme‐catalysed reactions.

## Fundamentals of enzyme‐based sensors

### http://link.springer.com/chapter/10.1007%2F1-4020-4611-1_16

This article defines and describes biosensors, including enzyme‐based systems.

## Enzyme technology: Biosensors

### http://www1.lsbu.ac.uk/water/enztech/biosensors.html

This site provides a primer on enzyme‐based sensors. It is a bit dated but still provides useful information.

## Highly sensitive glucose sensors

### http://www.nature.com/articles/srep08311?WT.ec_id=SREP-639-20150210

This article deals with enzyme‐based glucose sensors. Sensors for glucose have a large market because of the large demand to test blood glucose in medicine.

## Enzyme‐based electrochemical sensors

### http://www.azosensors.com/Article.aspx?ArticleID=42

This site provides a brief primer on enzyme‐based electrochemical sensors.

## OptiEnz Sensors

### http://optienz.com

This company constructs real‐time sensors for a variety of chemicals. The sensors are based on enzyme‐catalysed reactions that give an optical readout that is converted to an electrical signal.

## Food safety kits

### http://www.biooscientific.com/Food-Feed-Safety-Residue-Test-Kits

This company sells kits for detecting chemicals in foods. Some of the kits are imunologically based; others are based on detecting products of enzyme‐catalysed reactions.

## Stability of enzymes in sensors

### http://www.gwent.org/presentations/aetstab_08.pdf

This set of slides provides some useful basic information on stabilizing proteins for various commercial applications, including as biosensors.

## Pens writing with enzyme sensor “ink”

### http://ucsdnews.ucsd.edu/pressrelease/pens_filled_with_high_tech_inks_for_do_it_yourself_sensors

This news article discusses writeable materials for making sensors that detect chemicals *in situ*.

## Enzyme electrodes

### http://www.wsi.tum.de/Portals/0/Media/Lectures/20082/98f31639-f453-466d-bbc2-a76a95d8dead/BiosensorsBioelectronics_lecture6.pdf

This page at the Technical University at Munich discusses biosensor construction from a technical standpoint.

## FLIR: Threat detection

### http://www.flir.com/threatdetection/display/?id=63297

This company specializes in radiological, chemical and biological detection. Included in their products are enzyme‐based sensors.

## Enzyme sensors for detecting nerve agents

### https://pdfs.semanticscholar.org/50fb/7c9712763e53af32d1365322ccb8a2366e72.pdf

This paper describes the enzyme characteristics and construction of a sensor device for monitoring toxic organophosphate nerve agents.

## Enzymes for detecting abrin toxin

### http://www.ncbi.nlm.nih.gov/pubmed/25527549

This research article discusses a method for using enzymes in tandem for detecting abrine, a compound that co‐extracts with the toxic protein abrin that has been indicated to be a threat for food adulteration.

## Use of enzymes for detecting pesticides

### http://www.sciencedirect.com/science/article/pii/S0045653510011768

This review article discusses enzyme‐based technology for detecting pesticides, specifically organochlorine, organophosphate and carbamate classes.

## Strong growth predicted for biosensor market

### http://www.sensorsmag.com/specialty-markets/medical/strong-growth-predicted-biosensors-market-7640

This article provides a good overview of biosensor technology, describes key market sectors for these sensors and projects growth of over 10% a year for these products.

## Applied Enzyme Technology Ltd

### https://ec.europa.eu/research/biotechnology/bio-entrepreneurs/enzyme_technology_en.html

Applied Enzyme Technology specializes in enzyme stabilization and some of their expertise is directed at enzyme stabilization for sensor technology.

## Innovative Sensor Technology

### http://www.ist-usadivision.com

This company offers many kinds of sensors for sensing chemicals and environmental conditions. They have new enzyme‐based sensors for detecting glucose, lactate, glutamine and glutamate.

